# How AI-Related Task Complexity Shapes Innovative Work Behavior: A Coping Theory Perspective

**DOI:** 10.3390/bs15111467

**Published:** 2025-10-28

**Authors:** Hongyi Cai, Yuhui Ge, Heng Zhao

**Affiliations:** 1 Business School, University of Shanghai for Science and Technology, Shanghai 200093, China; gyh5688@usst.edu.cn; 2 School of Economics and Management, Beijing Jiaotong University, Beijing 100044, China; 23111225@bjtu.edu.cn

**Keywords:** AI-related task complexity, problem-focused coping, emotion-focused coping, AI opportunity perception, innovative work behavior

## Abstract

As technological revolutions continue to advance, AI increasingly emerges as a focal driver for enhancing innovation quality. Grounded in coping theory, this study develops a moderated dual-pathway model to examine the mechanisms through which AI-related task complexity influences innovative work behavior. A three-wave field survey was conducted among 353 employees from high-tech enterprises in Beijing and Shanghai. Hypotheses are tested via structural equation modeling. The findings reveal that AI-related task complexity significantly promotes innovative work behavior by fostering problem-focused coping while simultaneously suppressing it by triggering emotion-focused coping. Moreover, AI opportunity perception is found to moderate these relationships, strengthening the positive effect of problem-focused coping and attenuating the negative effect of emotion-focused coping on innovation. This study advances theoretical understanding of employee behavioral responses in AI-integrated work contexts and offers practical insights into how organizations can leverage AI to stimulate innovation among their workforce.

## 1. Introduction

In recent years, the remarkable development of AI has positioned it as a pivotal force behind advancements in science, industrial transformation, and enhanced productivity (Parteka & Kordalska, 2023). To secure a competitive edge, countries increasingly center their policies around AI advancement. Originally confined to production lines, AI now permeates services and high-value intellectual sectors, revolutionizing how businesses operate, leaders make decisions, and employees perform tasks (Budhwar et al., 2023; Savage, 2020). The widespread integration of AI is now considered an irreversible trend. In this context, innovation becomes a key driver for organizations aiming to maintain market leadership and pursue sustainable growth. Consequently, the question of how to harness AI-enabled opportunities to stimulate employee creativity has become a critical focus of both research and practice (Han et al., 2023).

Literature analysis indicates three dominant research strands in AI studies. The primary strand explores employee adoption readiness, principally employing the Technology Acceptance Model (TAM) and Unified Theory of Acceptance and Use of Technology (UTAUT) (Kim et al., 2024; Xia & Chen, 2024). The second stream draws on cognitive appraisal theory and the Job Demands-Resources (JD-R) model to explore how factors such as AI awareness, usage frequency, and AI-related anxiety influence employee behavior (Arboh et al., 2025; Chuang & Huang, 2025; Dong et al., 2024b; Nguyen et al., 2025; Radic et al., 2024). The third stream centers on human-AI collaboration, emphasizing how AI tools enhance task efficiency and employee creativity (Przegalinska et al., 2025; Wu & Zhang, 2024; J. Q. Xu et al., 2025). Although these investigations establish a conceptual framework for comprehending AI’s organizational impact, they predominantly characterize AI as either an exogenous asset or auxiliary instrument, neglecting the operational stressors and cognitive demands practitioners encounter when implementing AI systems. In reality, although AI offers considerable advantages, it inevitably imposes greater learning demands and skill updating pressures on employees, intensifying work complexity, particularly AI-related task complexity (Passalacqua et al., 2025; Teng et al., 2023). This complexity primarily manifests in the difficulty of the tasks themselves, the cognitive load required, and the problem-solving abilities needed when employees engage in AI-related work (Dong et al., 2024a). Despite the growing prevalence of AI-integrated work, limited research has addressed how AI-related task complexity influences employees’ innovative work behavior.

Drawing from a notable gap in the existing literature, this study aims to utilize coping theory as a lens to examine how employees respond to AI-related task complexity. Coping theory suggests that when individuals perceive external situations as challenging or uncertain, they typically adopt various coping strategies to regulate their emotional states and achieve psychological adjustment. These strategies are generally categorized into two types: emotion-focused coping and problem-focused coping. Emotion-focused coping is passive, primarily aimed at alleviating stress through emotional responses, avoidance, or self-absorption (Billings & Moos, 1984), while problem-focused coping is active, emphasizing the evaluation of the current situation and taking action to solve difficulties (Achnak & Vantilborgh, 2021; Badi, 2023; Pearsall et al., 2009). As AI becomes increasingly integrated into organizational work, employees often perceive challenges, such as increased task ambiguity and heightened skill requirements, which may in turn trigger a variety of psychological responses. What appears particularly significant about these findings is that problem-focused coping seems to entail initiating targeted measures to confront stress triggers, such as engaging in active learning, while emotion-focused coping ostensibly aims to alleviate internal emotional distress, often through avoidance (Lansisalmi et al., 2000; Pearsall et al., 2009). This indicates that by examining these coping strategy pairs, this study seeks to uncover how AI-related task complexity influences innovative work behavior, thereby providing theoretical support for understanding its underlying mechanisms.

In addition, AI opportunity perception is introduced as a key boundary condition, considering that employees’ coping responses to task complexity are substantially influenced by a range of individual differences. From this particular interpretive perspective, AI opportunity perception refers to employees’ subjective recognition of the potential benefits that may be brought by AI, such as career development, skill enhancement, and work optimization (Brougham & Haar, 2018; G. Xu et al., 2023). This perception reflects an underlying openness to technological change and a growth-oriented mindset. Individuals with a heightened perception of AI opportunity tend to focus on technological affordances and are more likely to view workplace transformations as potential career opportunities rather than threats (Grundner & Neuhofer, 2021). It follows that as AI continues to reshape the nature of work, such perceptual differences will influence how employees make sense of task complexity and, thus, shape their ultimate choice of coping strategies.

This study makes several contributions to the existing literature. First, it extends the focus of AI research to a deeper level by examining AI-related task complexity and its influence on employees’ innovative behavior. By investigating how AI integration shapes specific task characteristics, this study advances our understanding of the workplace realities of human–AI collaboration. Second, the study emphasizes employees’ concrete coping strategies and applies coping theory to uncover the underlying mechanism between AI-related task complexity and innovative behavior. It explicitly identifies problem-focused coping and emotion-focused coping as the key mediating mechanisms linking AI-related task complexity to employees’ innovative work behavior. Finally, the study introduces AI opportunity perception as a critical moderating variable, revealing how individuals cognitively evaluate the balance between pressure and opportunity and subsequently choose their coping responses. Together, these insights provide a more comprehensive theoretical framework for understanding how AI-related task complexity shapes employee innovation within AI integrated work environments.

## 2. Theory and Hypotheses

### 2.1. The Double-Edged Effect of AI-Related Task Complexity on Innovative Work Behavior

#### 2.1.1. The Mediating Role of Problem-Focused Coping

AI-related task complexity refers to the perceived operational challenges employees encounter when working with AI systems, which are reflected in factors such as task unpredictability, substantially elevated knowledge demands, and coordination mediated by technology (Dong et al., 2024a; Verma & Singh, 2022). Given the multifaceted nature of this evidence, as AI progressively permeates organizational workflows, employees are typically required to manage increasingly complex task scenarios involving data analysis, task reconfiguration, and human-AI collaboration (Nawaz et al., 2024). In light of these methodological considerations, employees must allocate greater cognitive resources to interpret task objectives, reframe execution logic, and resolve technical challenges. According to coping theory, when employees perceive task complexity as a developmental challenge rather than a threat, they are more likely to employ problem-focused coping strategies, such as actively seeking information or adjusting operational approaches (Nasaj et al., 2025; Pearsall et al., 2009). What also appears significant in this context is how the dynamic and iterative nature of AI-related tasks seems to lend support to what may represent behavioral adaptation and cognitive alignment, potentially increasing the likelihood of proactive coping responses (Q. Zhou et al., 2024). Therefore, this research proposes the following hypothesis:

**Hypothesis** **1a.** *AI-related task complexity will be positively related to problem-focused coping.*

Innovative work behavior refers to the process through which employees proactively generate, promote, and implement novel ideas in their work. Achieving innovation depends not only on the accumulation of knowledge and resources but also on individuals’ coping tendencies when facing complex tasks (Janssen, 2000). Problem-focused coping, as a proactive strategy, motivates employees to identify problems, integrate information, and adjust strategies to seek optimal solutions under uncertain conditions (Badi, 2023). When employees adopt problem-focused coping to address complex tasks, they tend to exhibit stronger intrinsic motivation and a greater sense of control, which enhances their confidence and willingness to explore new possibilities (Lansisalmi et al., 2000; Pearsall et al., 2009; Yin et al., 2024). Particularly in tasks involving AI, feedback in real time and predictive insights provided by AI systems further strengthen employees’ learning and continuous improvement behaviors, thereby enhancing their problem solving and creative capabilities (Q. Zhou et al., 2024). Therefore, the more actively employees engage in problem focused coping, the more likely they are to demonstrate sustained innovative work behavior. Therefore, this research proposes the following hypothesis:

**Hypothesis** **1b.** *Problem-focused coping will be positively related to innovative work behavior.*

The restructuring of work brought about by artificial intelligence not only increases task complexity but also generates greater opportunities for employee development and creativity. When employees adopt problem focused coping strategies in response to task complexity arising from the use of AI, they are more likely to transform these challenges into sources of innovation through active learning, information integration, and behavioral adjustment. Within this process, employees are released from repetitive tasks, allowing them to generate and advance new ideas in complex environments and to concentrate on more challenging and creative activities (Arias-Pérez & Vélez-Jaramillo, 2022). Therefore, this research proposes the following hypothesis:

**Hypothesis** **1c.** *AI-related task complexity will be positively related to innovative work behavior through problem-focused coping.*

#### 2.1.2. The Mediating Role of Emotion-Focused Coping

Emotion-focused coping involves changing one’s thoughts or feelings about the stressful situation, rather than taking direct action, in order to alleviate related negative emotions (Nasaj et al., 2025; Vaziri et al., 2020). As AI becomes increasingly embedded within organizational operations, employees face not only changes in task structure and skill requirements but also shifts in role perception and fluctuations in their psychological states. When AI-related task complexity is perceived as high, especially when employees feel underqualified, lack sufficient support, or experience uncertainty, emotional responses such as tension, helplessness, or anxiety are likely to be triggered (Stein et al., 2020; Yin et al., 2024). Under such circumstances, employees are more inclined to adopt emotion-focused coping strategies, such as temporarily avoiding stressors, venting emotions, or reducing engagement, in an effort to alleviate negative feelings and restore psychological balance. Therefore, the stronger the perceived AI-related task complexity, the more likely employees are to engage in emotion-focused coping behavior. Therefore, this research proposes the following hypothesis:

**Hypothesis** **2a.** *AI-related task complexity will be positively related to emotion-focused coping.*

Innovative work behavior requires substantial cognitive engagement and is supported by positive emotional states (Amabile & Pratt, 2016). However, emotion-focused coping primarily aims to regulate negative emotions and is often manifested through avoidance, suppression, problem minimization, or reliance on external comfort (George & Zhou, 2007). Although such coping may alleviate psychological stress in the short term, it undermines employees’ proactivity and engagement in the face of complex tasks, weakening their motivation for continuous learning and deep thinking (Carver et al., 1989; Gross, 1998).

From a behavioral perspective, emotion-focused coping diverts employees’ attention toward emotional regulation rather than problem solving, resulting in diminished exploratory behavior and reduced innovation when facing challenges (Baumeister et al., 2007; Carver et al., 1989). Moreover, emotion-focused strategies are often associated with feelings of exhaustion, loss of control, and cognitive closure, all of which impair the ability to generate new ideas and drive sustained change in highly demanding tasks (Halbesleben & Bowler, 2007; Tourigny et al., 2013). These findings suggest that emotion-focused coping exerts a significant negative influence on innovative work behavior. Therefore, this research proposes the following hypothesis:

**Hypothesis** **2b.** *Emotion-focused coping will be negatively related to innovative work behavior.*

Although AI introduces new momentum for organizational development, the complexity it brings to tasks may not always elicit positive responses. For some employees, AI-related task complexity increases learning anxiety and raises concerns about insufficient competence and unclear role boundaries (Schiavo et al., 2024; Y. M. Wang et al., 2024; Wu et al., 2024). When such complexity is primarily managed through emotional regulation rather than constructive action, employees may struggle to adapt effectively to task demands and are less likely to generate sustained ideas or innovative solutions. Therefore, this research proposes the following hypothesis:

**Hypothesis** **2c.** *AI-related task complexity will be negatively related to innovative work behavior through emotion-focused coping.*

### 2.2. The Moderating Role of AI Opportunity Perception

AI opportunity perception refers to the extent to which employees view artificial intelligence as a positive resource that promotes personal growth and career development. When facing AI-related task complexity, employees’ subjective evaluations of the task significantly influence their coping responses (Lazarus, 1991). Employees with high AI opportunity perception are more likely to interpret complex tasks as opportunities for breakthrough and tend to engage in deeper reflection, problem-solving, and skill development (Amabile & Pratt, 2016; Fredrickson, 2001). For these individuals, task complexity is perceived as a source of learning and challenge, which motivates the adoption of problem-focused coping strategies and enhances both their sense of control and intrinsic motivation (Ohly & Fritz, 2010; Sonnentag, 2003). From this interpretive perspective, employees with a low perception of AI opportunities are more likely to focus on the stress and uncertainty associated with technology. This focus reduces their willingness to cope and may lead them to avoid challenging tasks, which in turn weakens the likelihood of adopting problem focused coping strategies (Brougham & Haar, 2018). These findings indicate that AI opportunity perception strengthens the positive relationship between AI-related task complexity and problem focused coping. Moreover, as problem focused coping serves as a key mechanism that facilitates innovative work behavior, its mediating effect becomes more pronounced when AI opportunity perception is high. Therefore, this research proposes the following hypothesis:

**Hypothesis** **3.** *AI opportunity perception will strengthen the positive relationship between AI-related task complexity and problem-focused coping.*

**Hypothesis** **4.** *AI opportunity perception will strengthen the mediating effect of problem-focused coping on the relationship between AI-related task complexity and innovative work behavior.*

Alternatively, employees with high AI opportunity perception tend to focus more on the positive potential of technological change (G. Xu et al., 2023). They are more likely to view AI-related tasks as challenges rather than threats, which reduces the likelihood of activating emotion-focused coping strategies in stressful situations. Individuals with high AI opportunity perception are better equipped for rational information processing and emotional regulation, thereby decreasing the occurrence of unconstructive reactions such as avoidance and suppression (Amabile & Pratt, 2016). In contrast, when employees lack awareness of the empowering potential of AI, uncertainty amplifies emotional fluctuations, leading them to rely on emotional relief strategies when confronting complex tasks. Consequently, their willingness and capacity to address the problem itself are weakened (Vaziri et al., 2020).

Furthermore, differences in emotional regulation capacity may determine whether emotion-focused coping continues to impair employees’ motivation for innovation. High AI opportunity perception helps reduce both the frequency and intensity of negative emotional strategies at the source, thereby diminishing their suppressive impact on innovative behavior. Therefore, this research proposes the following hypothesis:

**Hypothesis** **5.** *AI opportunity perception will weaken the positive relationship between AI-related task complexity and emotion-focused coping.*

**Hypothesis** **6.** *AI opportunity perception will weaken the mediating effect of emotion-focused coping on the relationship between AI-related task complexity and innovative work behavior.*

**Figure 1:**
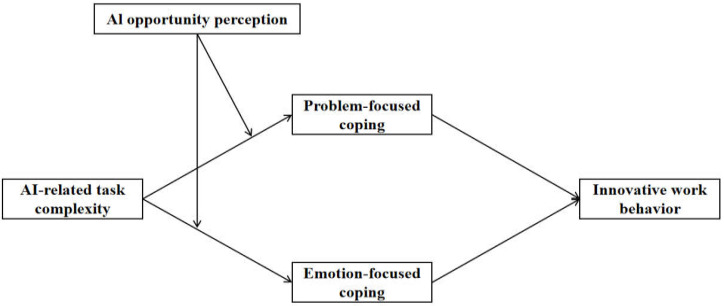
Theoretical model.

The theoretical model of this study is shown in Figure 1.

## 3. Research Methodology

### 3.1. Research Procedure and Sample

Data collection was conducted via the web-based survey tool Credamo to gather questionnaire responses. Participants were recruited from five companies located in Beijing and Shanghai, covering the service and internet industries. With the assistance of each company’s human resources department, employees were invited to participate voluntarily, and they were guaranteed that their responses would be kept strictly confidential. Compared to cross-sectional data, longitudinal studies with multiple time points can help reduce common method bias (Podsakoff et al., 2003). Therefore, this study adopted a three-wave time-lagged design, with a two-week interval between each wave. In the first wave (T1), 487 questionnaires were distributed with 442 valid responses returned, achieving a response rate of 90.760%. These collected data covered AI-related task complexity, Al opportunity perception and demographic information. The second wave (T2) then focused on gathering data regarding both problem-focused and emotion-focused coping strategies. Of the 442 questionnaires distributed, 388 valid responses were obtained, resulting in a response rate of 87.783%. The third wave (T3) data collection specifically targeted second-wave respondents, requiring them to self-assess their innovative work behavior manifestations. Among the 388 questionnaires distributed, 353 valid responses were retained after removing patterned responses and unmatched identifiers, resulting in a final response rate of 90.979%. Regarding the demographic characteristics of the final sample, 51.27% of respondents were male and 48.73% were female. In terms of age, 11.33% were under 20 years old, 27.48% were between 21 and 30, 45.89% between 31 and 40, and 15.30% were over 40. For educational background, 35.98% held an associate degree or below, 45.04% held a bachelor’s degree, 14.45% held a master’s degree, and 4.53% held a doctoral degree or above. As for work experience, 16.43% had fewer than 3 years, 14.73% had 3–5 years, 34.28% had 6–8 years, 19.83% had 9–10 years, and 14.73% had more than 10 years. The participant profile is provided in Appendix A.

### 3.2. Measurement Tools

To assess both reliability and validity of the measurement instrument, the measurement scales for all study variables, including Al-related task complexity, problem focused coping, emotion focused coping, AI opportunity perception, and innovative work behavior, are adapted from well-established scales published in leading international journals. The research team implemented standardized translation-back translation protocols to adapt all measurement items into Chinese. All constructs were assessed via five-point Likert scales anchored at 1 (strongly disagree) to 5 (strongly agree).

AI-related task complexity: A 4-item scale developed by Dong et al. (2024a) was utilized to assess AI-related task complexity. Example items include statements like, “I found the task of working with AI to be a complex task.” The scale demonstrated high reliability, with a Cronbach’s α of 0.883.

Problem-focused coping: A 4-item scale developed by Patzelt and Shepherd (2011) was used to measure problem-focused coping. Example items include statements like, “I try to look at the situation from a different angle.” The scale demonstrated adequate reliability, with a Cronbach’s α of 0.836.

Emotion-focused coping: A 6-item scale developed by Patzelt and Shepherd (2011) was used to assess emotion-focused coping. Example items include statements like, “I drink alcohol or take medication to feel better.” The scale demonstrated high reliability, with a Cronbach’s α of 0.916.

AI opportunity perception: A 5-item scale adapted from Highhouse and Yüce (1996) was used to measure AI opportunity perception. Example items include statements like, “I believe that the application of AI in the organization increases the likelihood of success in my career development.” The scale demonstrated high reliability, with a Cronbach’s α of 0.922.

Innovative work behavior: A 6-item scale developed by Scott and Bruce (1994) was utilized to assess innovative work behavior. Example items include statements like, “I frequently use new processes, technologies, or methods in my work.” The scale demonstrated high reliability, with a Cronbach’s α of 0.911.

Control Variables: Because individual demographic characteristics may influence research outcomes (Spector & Brannick, 2010), gender, age, education level, and years of work experience were treated as control variables in this study.

### 3.3. Analytic Strategy

The statistical analyses were conducted using SPSS 26, AMOS 28 and Mplus 8.3. First, SPSS 26 and AMOS 28 were used to perform descriptive statistics as well as reliability and validity assessments. Reliability was evaluated by calculating Cronbach’s α, composite reliability (CR), and average variance extracted (AVE) to assess the internal consistency and reliability of the scales. Subsequently, exploratory factor analysis (EFA) was conducted to examine the structural validity of the scales, and the square roots of the AVE were used to assess discriminant validity. Next, confirmatory factor analysis (CFA) was performed using AMOS 28 to verify the consistency between the latent factor structure and the observed data. Model fit was evaluated using multiple indices, including RMSEA, CFI, GFI, IFI, and TLI, to determine the adequacy of model fit. Finally, structural equation modeling (SEM) was applied to test the hypothesized relationships. SEM enables the simultaneous estimation of relationships among latent variables and allows for the examination of both mediating and moderating effects. Through Mplus 8.3, the overall path model was constructed to systematically test the proposed hypotheses. The detailed empirical results are presented in the following sections.

## 4. Results

### 4.1. Reliability and Validity Testing

Table 1 presents the results of the reliability and convergent validity analyses for the main variables of this study. All variables have CR and Cronbach’s alpha coefficients exceeding the recommended threshold of 0.70, indicating good internal consistency of the scales. In addition, all standardized factor loadings are above the acceptable standard of 0.70, and the AVE values are greater than 0.50, demonstrating that each construct exhibits satisfactory convergent validity.

This study conducted EFA to validate the questionnaire’s effectiveness. The results showed a Kaiser-Meyer-Olkin (KMO) measure of 0.909 (exceeding the recommended threshold of 0.8), and Bartlett’s Test of Sphericity was significant (Approximate Chi-square = 5708.399, df = 300, *p* < 0.001), indicating sufficient correlations for factor analysis. Principal component analysis was employed with varimax rotation, extracting five factors with eigenvalues greater than 1. As shown in Table 2, these factors explained variance proportions of 17.125%, 16.701%, 15.366%, 11.978%, and 10.552%, respectively, with a cumulative variance of 71.722% (exceeding the 50% acceptability threshold), demonstrating effective representation of the original variables’ information.

Table 3 presents the means, standard deviations, correlation coefficients and discriminant validity for all variables in the model, with the bolded values on the diagonal representing the square roots of the AVE for each core variable. Discriminant validity was assessed using the Fornell-Larcker criterion, which showed that the square root of the AVE for each core variable was greater than its correlations with all other core variables, supporting adequate discriminant validity. Further analysis of variable relationships revealed that the correlations between the demographic control variables and the five core variables were not statistically significant (*p* > 0.05). AI-related task complexity was positively correlated with innovative work behavior (r = 0.240, *p* < 0.01), as well as with problem-focused coping (r = 0.225, *p* < 0.01) and emotion-focused coping (r = 0.192, *p* < 0.01). Moreover, problem-focused coping showed a relatively strong positive correlation with innovative work behavior (r = 0.466, *p* < 0.01), while emotion-focused coping was negatively correlated with innovative work behavior (r = −0.283, *p* < 0.01). These results provide preliminary support for the hypothesized relationships among the core variables.

**Figure 2:**
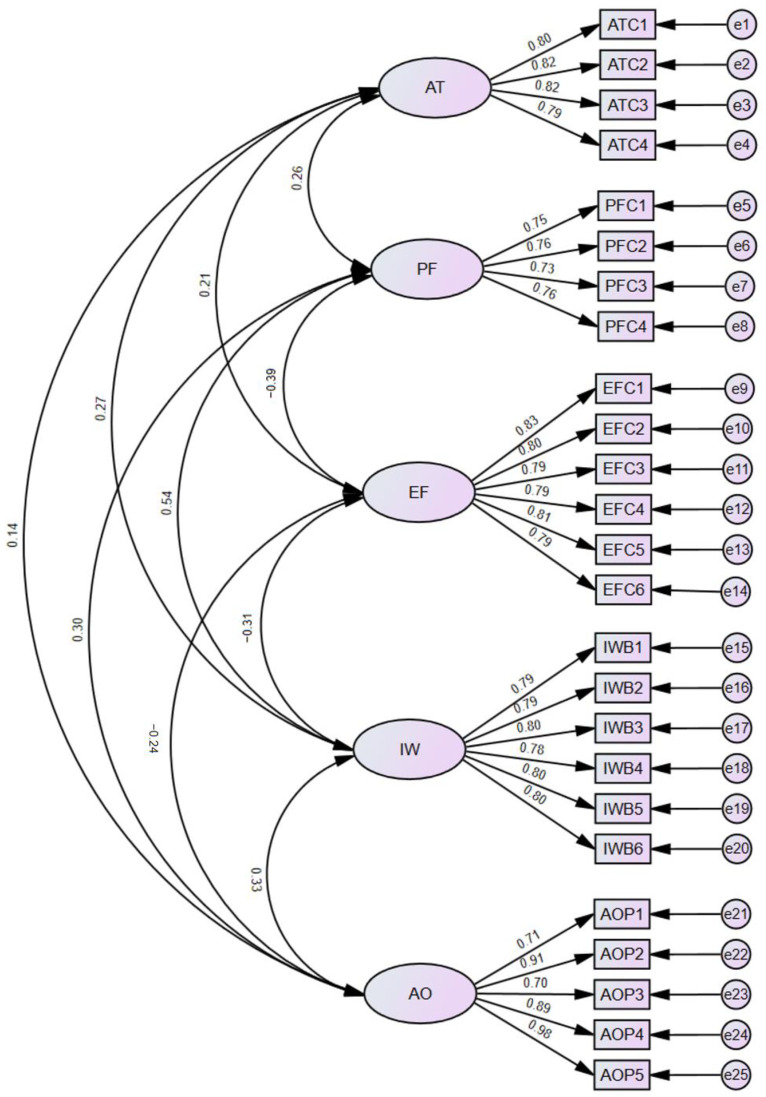
Confirmatory factor analysis model diagram.

Discriminant validity was assessed through CFA, with competing measurement models being compared to evaluate model fit indices. The five-factor model is specified as the baseline model, and four competing models are constructed: a four-factor model (combining AI-related task complexity and AI opportunity perception), a three-factor model (combining AI-related task complexity, problem-focused coping, and AI opportunity perception), a two-factor model (combining AI-related task complexity, emotion-focused coping, problem-focused coping, and AI opportunity perception), and a one-factor model (combining all variables into a single factor) (see Table 4). The corresponding measurement model is presented in Figure 2. The results show that the five-factor model demonstrates the best model fit (χ^2^/df = 0.985, RMSEA = 0.002, CFI = 0.993, GFI = 0.945, IFI = 0.994, TLI = 0.992), indicating good discriminant validity for the measurement model in this study.

### 4.2. Hypothesis Testing

**Figure 3:**
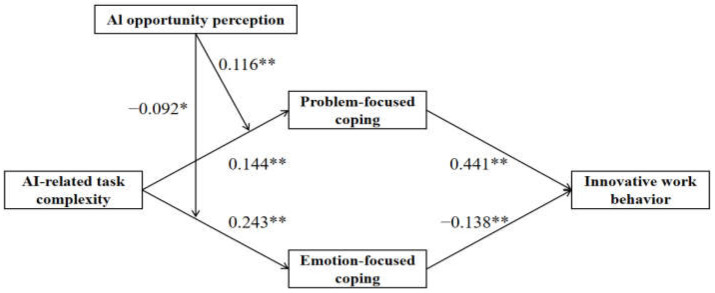
Results of path coefficient analysis. Note: ** *p* < 0.01, * *p* < 0.05.

Path analysis was employed to disentangle the complex relationships among multiple variables, particularly those involving multiple mediators and moderators (L. Wang & Preacher, 2015). Accordingly, this study utilized Mplus 8.3 to conduct a SEM analysis. The results of this analysis are presented in Figure 3. All hypothesized relationships were tested while controlling for relevant variables. It was found that the control variables had no significant impact on the core constructs in this model. The path coefficients for the main research variables, derived from the structural equation model, are reported in Table 5.

As shown in Table 5, AI-related task complexity is found to be positively associated with problem-focused coping (B = 0.144, *p* < 0.01), providing support for H1a. AI-related task complexity is also positively associated with emotion-focused coping (B = 0.243, *p* < 0.01), supporting H2a. In addition, problem-focused coping is positively associated with innovative work behavior (B = 0.441, *p* < 0.01), supporting H1b, while emotion-focused coping is negatively associated with innovative work behavior (B = −0.138, *p* < 0.01), supporting H2b.

To further examine the mediating effects, we performed a bootstrap analysis with 5000 samples and 95% bias-corrected confidence intervals (CI). The results, presented in Table 6, indicate that, AI-related task complexity exerts a significant positive indirect effect on innovative work behavior through problem-focused coping, with a mediation effect of 0.063 (SE = 0.023, 95% CI [0.020, 0.111]), supporting H1c. Simultaneously, AI-related task complexity has a significant negative indirect effect on innovative work behavior via emotion-focused coping, with a mediation effect of −0.034 (SE = 0.013, 95% CI [−0.062, −0.012]), supporting H2c. Since both confidence intervals do not include zero, the mediating effects are considered statistically significant. These findings suggest a double-edged effect of AI-related task complexity on innovative work behavior: on one hand, it enhances innovation by promoting problem-focused coping; on the other hand, it may inhibit innovation by increasing reliance on emotion-focused coping.

**Figure 4:**
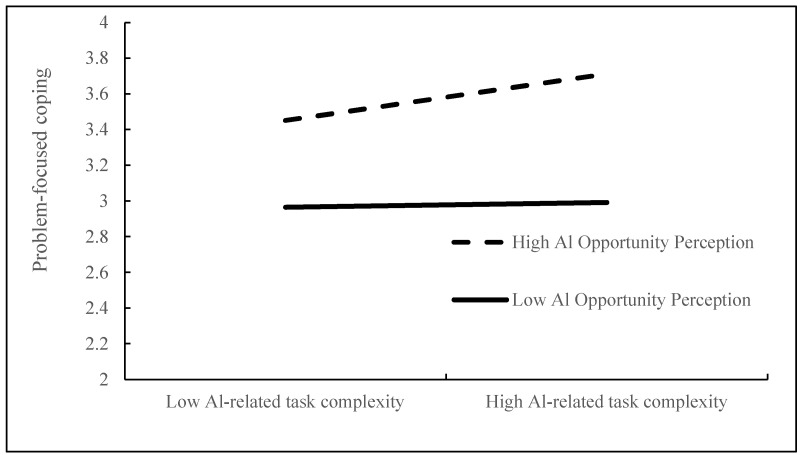
Moderating effect of AI opportunity perception on problem-focused coping.

**Figure 5:**
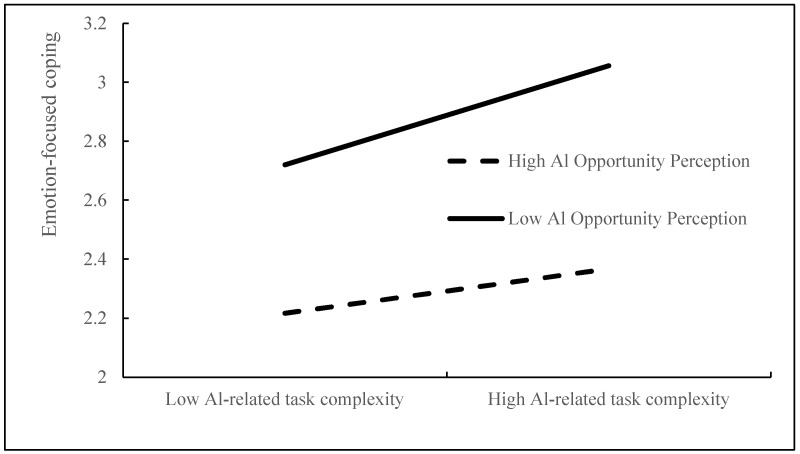
Moderating effect of AI opportunity perception on emotion-focused coping.

The moderation effect tests (see Table 5) reveal that the interaction between AI-related task complexity and AI opportunity perception exerts a significant positive influence on problem-focused coping (B = 0.116, *p* < 0.01), supporting H3, while a significant negative influence on emotion-focused coping (B = −0.092, *p* < 0.05) supports H5. To further probe these interactions, simple slope analyses were conducted, as illustrated in Figure 4 and Figure 5.

Table 7 displays the moderated mediation analysis outcomes. The indirect pathway from AI-related task complexity to innovative behavior through problem-focused coping demonstrates significant moderation by AI opportunity perception. When AI opportunity perception is high, the indirect effect is significant (Effect = 0.114, Boot 95% CI [0.057, 0.182]); when it is low, the effect becomes nonsignificant (Effect = 0.012, Boot 95% CI [−0.046, 0.073]). These findings suggest that higher levels of AI opportunity perception strengthen the positive indirect pathway, providing support for H4. Furthermore, AI opportunity perception significantly moderates the adverse mediation pathway whereby AI-related task complexity diminishes innovative behavior through emotion-focused coping. Specifically, when AI opportunity perception is low, the indirect effect is significantly negative (Effect = −0.046, Boot 95% CI [−0.087, −0.016]); this negative effect is attenuated when AI opportunity perception is high (Effect = −0.021, Boot 95% CI [−0.050, −0.003]). These results indicate that AI opportunity perception mitigates the adverse impact of emotion-focused coping on innovative behavior, thereby supporting H6.

## 5. General Discussion

Based on coping theory, this study develops a moderated mediation model to investigate the mechanism through which AI-related task complexity influences employees’ innovative work behavior. Utilizing a three-wave time-lagged research design and analyzing data from 353 valid responses, the empirical results support the proposed moderated mediation model. The findings reveal that while AI-related task complexity can stimulate employees to adopt problem-focused coping strategies, it may also trigger emotion-focused coping, resulting in a dual-effect on innovative work behavior. Furthermore, the study demonstrates that AI opportunity perception plays a crucial moderating role in these pathways. When employees perceive higher levels of opportunities related to AI, it not only strengthens the positive effect of problem-focused coping on innovative work behavior but also effectively mitigates the negative impact of emotion-focused coping. On this basis, the discussion proceeds in three key aspects: theoretical contributions, practical implications, and research limitations.

### 5.1. Theoretical Contributions

First, this study addresses a gap in the existing literature on AI and job design by focusing on the task-level impact of AI systems. Prior research in organizational behavior primarily emphasizes employees’ adaptation to and adoption of AI technologies, such as AI adoption (Cheng et al., 2023; Quan et al., 2025), human-AI collaboration (J. Q. Xu et al., 2025), and AI usage behaviors (X. Zhou et al., 2025), often highlighting the positive or negative outcomes of AI-enabled tools on performance and learning (Sun et al., 2025; Wu & Zhang, 2024; Q. Zhou et al., 2024). However, less attention has been given to how AI integration may increase task complexity, thereby eliciting diverse psychological responses and behavioral outcomes. At the same time, traditional job design and complexity research primarily focuses on general task demands and rarely explores how task complexity shaped by AI affects employees’ innovative work behavior.

Second, this study advances the understanding of employees’ psychological dynamics in AI-integrated work environments by uncovering two parallel coping pathways: problem-focused coping and emotion-focused coping. Prior research on job demands and employee behavior, often grounded in the stressor framework or the job demands-resources model (Bakker & Demerouti, 2017; Cheng et al., 2023; Dong et al., 2024b), primarily focuses on the attributes of job demands or their interactions with available resources. However, relatively little attention has been given to how employees adopt diverse coping strategies based on their subjective perceptions when facing the same task demands. Moreover, some studies tend to overlook the underlying psychological mechanisms involved. This study emphasizes that AI-related task complexity in real world settings can simultaneously trigger both positive and negative coping responses. We distinctly reveal the parallel operation of problem-focused and emotion-focused coping mechanisms while delineating their differential impacts on innovative work behavior. These theoretical advancements extend the relevance of coping theory to digital work environments and elucidate the cognitive and emotional processes underlying employee adaptation to task complexity caused by AI.

Finally, this study incorporates AI opportunity perception as a boundary condition, highlighting how employees’ positive appraisals of AI contexts shape the link between task complexity, coping strategies, and behavioral outcomes. While existing research primarily emphasizes the threats and job displacement risks posed by AI (Bai et al., 2025; Demirci et al., 2025), little is known about how employees’ perceptions oriented toward opportunities help reframe task complexity and activate personal potential. This study finds that high levels of AI opportunity perception significantly strengthen the positive effect of problem-focused coping on innovative work behavior while simultaneously buffering the negative effect of emotion-focused coping. These insights enhance the contextual sensitivity of coping theory and offer a theoretical foundation for understanding how employees empower themselves through positive cognitive framing in the face of ongoing AI adoption.

### 5.2. Practical Implications

First, as AI technologies are increasingly integrated into organizational routines, employees are exposed to rising levels of task complexity and uncertainty. This study finds that AI related task complexity can act as a challenging demand that encourages proactive coping, but it may also generate additional cognitive burdens and trigger emotional avoidance, which ultimately influence innovative work behavior. Therefore, managers should carefully consider both the operability and cognitive load when designing AI applications. Instead of excessively stacking tasks, it is essential to account for employees’ capacity to absorb and adapt. Strategies such as task decomposition, clear goal setting, and periodic feedback can assist employees in gradually adapting to the complexity introduced by AI and framing it as a developmental challenge that supports sustained innovation.

Second, the coping strategies that employees adopt when responding to AI related task demands are essential for enabling innovative work behavior. Organizations that foster problem focused coping are more likely to support the full realization of employees’ creative potential. Practical measures include providing emotional regulation and psychological capital training, encouraging cross departmental knowledge exchange, and establishing peer coaching and mentoring mechanisms. Timely support and responsive assistance in dealing with AI challenges help reduce anxiety and strengthen creative engagement.

Finally, managers are encouraged to communicate the connection between AI capabilities and career development clearly. Emphasizing the role of AI skills in enhancing job competence and expanding future opportunities can help employees form a positive view of technological transformation. Organizations can support this process through initiatives such as internal knowledge sharing, career path mapping, AI training certifications, and cross functional job rotation. These efforts help employees build stronger self-efficacy and adaptability, allowing them to respond more effectively to AI related complexity and unlock their innovative potential.

### 5.3. Limitations and Future Research

First, although a multi-wave survey design is adopted and both reliability and validity of key constructs are tested, the data are primarily derived from employees’ self-reports of AI-related task complexity, coping strategies, and innovative work behavior. While such self-reported data are useful for capturing psychological perceptions and behavioral tendencies, they may also be subject to individual cognitive biases and social desirability effects. Future studies are encouraged to incorporate multi-source data for triangulation, such as actual AI usage logs, project assignment records, or third-party ratings of innovative behavior by supervisors and coworkers, to enhance the accuracy and credibility of the findings.

Second, this study adopts an individual level perspective to analyze how AI related task complexity impacts innovative work behavior through two pathways: problem focused and emotion focused coping. However, actual organizational contexts show employee coping patterns develop within multilayered environments influenced by organizational support climate, leadership behaviors, and organizational culture. For example, employees’ perceptions of challenge and opportunity posed by AI tasks may vary depending on the AI adoption climate within teams or the attitudes of supervisors and peers. Thus, future research could incorporate multilevel or team level contextual variables to conduct cross-level analyses, such as developmental feedback from leaders or a shared learning climate, to test potential moderating effects and improve the applicability of findings in complex organizational contexts.

Finally, beyond coping strategies, employees’ responses to AI-related tasks may also be jointly influenced by individual differences, institutional support systems, and career development stages. For example, factors such as self-efficacy, digital literacy, and growth mindset may critically affect how employees cope with AI tasks and engage in innovation. In addition, future studies could expand sample sources or examine different industry contexts to explore whether the effects of AI-related task complexity on innovative work behavior vary significantly across settings (Podsakoff et al., 2003). Such research endeavors delineate the fundamental mechanisms underlying employee innovation in workplaces enhanced by AI and to offer actionable insights for optimizing talent development and technology management.

## 6. Conclusions

Today, artificial intelligence is widely applied across industries and is gradually evolving from an auxiliary tool to a collaborative partner that works alongside human employees, referred to as human–AI collaboration. Within this emerging work model, this study focuses on the impact of AI-related task complexity on employees’ innovative behavior, drawing on coping theory to uncover its underlying mechanism and examining the boundary effect of AI opportunity perception. The results indicate that AI task complexity can promote innovative behavior by stimulating problem-focused coping, but it may also hinder innovation by triggering emotion-focused coping. Furthermore, AI opportunity perception strengthens the positive effect of problem-focused coping while mitigating the negative impact of emotion-focused coping. These findings provide valuable theoretical and practical insights for organizations seeking to enhance employee innovation in the context of deep AI integration.

## Figures and Tables

**Table 1:** Validity and reliability of items.

Variables	Items	Standardized Factor Loadings	AVE	CR	Cronbach’s α
Al-related task complexity	ATC1	0.797	0.653	0.883	0.883
	ATC2	0.820			
	ATC3	0.822			
	ATC4	0.793			
Problem-focused coping	PFC1	0.751	0.56	0.836	0.836
	PFC2	0.756			
	PFC3	0.729			
	PFC4	0.755			
Emotion-focused coping	EFC1	0.830	0.645	0.916	0.916
	EFC2	0.798			
	EFC3	0.792			
	EFC4	0.792			
	EFC5	0.812			
	EFC6	0.793			
Innovative work behavior	IWB1	0.790	0.63	0.911	0.911
	IWB2	0.790			
	IWB3	0.799			
	IWB4	0.778			
	IWB5	0.804			
	IWB6	0.801			
Al Opportunity Perception	AOP1	0.710	0.714	0.924	0.922
	AOP2	0.908			
	AOP3	0.699			
	AOP4	0.889			
	AOP5	0.979			

**Table 2:** Exploratory Factor Analysis Results.

Variables	Codes	Rotated Factor Loadings					KMO	Bartlett’s Test of Sphericity		
		1	2	3	4	5		Approximate Chi-Squared Value	df	*p*
Al-related task complexity	ATC1	0.836					0.834	738.663	6	0.000
	ATC2	0.833								
	ATC3	0.852								
	ATC4	0.828								
Problem-focused coping	PFC1		0.789				0.816	517.49	6	0.000
	PFC2		0.731							
	PFC3		0.758							
	PFC4		0.776							
Emotion-focused coping	EFC1			0.835			0.924	1311.845	15	0.000
	EFC2			0.808						
	EFC3			0.803						
	EFC4			0.827						
	EFC5			0.820						
	EFC6			0.799						
Innovative work behavior	IWB1				0.785		0.923	1243.916	15	0.000
	IWB2				0.791					
	IWB3				0.799					
	IWB4				0.777					
	IWB5				0.823					
	IWB6				0.790					
Al Opportunity Perception	AOP1					0.770	0.864	1524.177	10	0.000
	AOP2					0.890				
	AOP3					0.773				
	AOP4					0.878				
	AOP5					0.937				
Overall Scale							0.909	5708.399	300	0.000
Eigen Value		2.995	2.638	4.281	4.175	3.841				
Explained Variance (%)		11.978	10.552	17.125	16.701	15.366				
Cumulative Variance (%)		11.978	22.530	39.655	56.356	71.722				

**Table 3:** Means, Standard Deviations, Correlations, and Discriminant Validity.

Variables	M	SD	1	2	3	4	5	6	7	8	9
1. Gender	1.487	0.501									
2. Age	2.652	0.873	−0.039								
3. Edu	1.875	0.82	−0.094	0.169 **							
4. Tenure	3.017	1.263	−0.027	0.858 **	0.147 **						
5. ATC	3.251	1.045	−0.024	0.077	−0.006	0.030	**0.808**				
6. AOP	3.448	1.009	−0.021	0.103	−0.068	0.098	0.153 **	**0.845**			
7. PFC	3.295	0.947	−0.001	0.037	−0.01	0	0.225 **	0.292 **	**0.748**		
8. EFC	2.656	1.009	0.046	−0.023	0.062	−0.027	0.192 **	−0.228 **	−0.339 **	**0.803**	
9. IWB	3.324	0.998	−0.07	0.025	0.004	0	0.240 **	0.331 **	0.466 **	−0.283 **	**0.794**

Note: ATC = AI-related task complexity, AOP = AI opportunity perception, PFC = Problem-focused coping, EFC = Emotion-focused coping, IWB = Innovative work behavior. ** *p* < 0.01. The values in bold on the diagonal are the square roots of the AVE.

**Table 4:** Comparison of alternative models in confirmatory factor analysis.

Model	χ^2^/df	RMSEA	CFI	GFI	IFI	TLI
Five-factor model: ATC, AOP, PFC, EFC, IWB	0.985	0.002	0.993	0.945	0.994	0.992
Four-factor model: ATC + AOP, PFC, EFC, IWB	6.616	0.126	0.729	0.667	0.731	0.698
Three-factor model: ATC + AOP + PFC, EFC, IWB	5.886	0.118	0.762	0.668	0.763	0.737
Two-factor model: ATC + AOP + PFC + EFC, IWB	10.438	0.164	0.536	0.479	0.539	0.492
One-factor model: ATC + AOP + PFC + EFC + IWB	13.810	0.191	0.368	0.418	0.371	0.311

Note: ATC = AI-related task complexity, AOP = AI opportunity perception, PFC = Problem-focused coping, EFC = Emotion-focused coping, IWB = Innovative work behavior.

**Table 5:** Results of direct and moderating effect hypothesis testing.

Hypothesis	Path	Estimate	SE	Results
H1a	ATC → PFC	0.144 **	0.047	Supported
H1b	PFC → IWB	0.441 **	0.046	Supported
H2a	ATC → EFC	0.243 **	0.051	Supported
H2b	EFC → IWB	−0.138 **	0.046	Supported
H3	ATC × AOP → PFC	0.116 **	0.045	Supported
H5	ATC × AOP → EFC	−0.092 *	0.046	Supported

Note: ATC = AI-related task complexity, AOP = AI opportunity perception, PFC = Problem-focused coping, EFC = Emotion-focused coping, IWB = Innovative work behavior. ** *p* < 0.01, * *p* < 0.05.

**Table 6:** Mediation effect test results.

Hypothesis	Path	Effect	Boot SE	BootLLCI	BootULCI	Results
H1c	ATC → PFC → IWB	0.063	0.023	0.020	0.111	Supported
H2c	ATC → EFC → IWB	−0.034	0.013	−0.062	−0.012	Supported

Note: ATC = AI-related task complexity, PFC = Problem-focused coping, EFC = Emotion-focused coping, IWB = Innovative work behavior.

**Table 7:** Results of moderated mediation effect hypotheses testing.

Hypothesis	Mediator	Moderator Level	Effect	Boot SE	BootLLCI	BootULCI	Results
H4	PFC	Low AOP	0.012	0.029	−0.046	0.073	Supported
		Medium AOP	0.063	0.023	0.020	0.111	
		High AOP	0.114	0.033	0.057	0.182	
H6	EFC	Low AOP	−0.046	0.018	−0.087	−0.016	Supported
		Medium AOP	−0.034	0.013	−0.062	−0.012	
		High AOP	−0.021	0.012	−0.050	−0.003	

Note: AOP = AI opportunity perception, PFC = Problem-focused coping, EFC = Emotion-focused coping.

## Data Availability

The data used to support the findings of this study are available from the corresponding author upon request.

## Appendix A

**Table A1:** Participant profile.

Variables	Category	Frequency	Percentage (%)
Gender	Male	181	51.27
	Female	172	48.73
Age	Under 20 years	40	11.33
	21–30 years	97	27.48
	31–40 years	162	45.89
	Over 40 years	54	15.3
Education	Associate degree or below	127	35.98
	Bachelor’s degree	159	45.04
	Master’s degree	51	14.45
	Doctoral degree or above	16	4.53
Tenure	Under 3 years	58	16.43
	3–5 years	52	14.73
	6–8 years	121	34.28
	9–10 years	70	19.83
	Over 10 years	52	14.73
Total		353	100
